# From cheek swabs to consensus sequences: an A to Z protocol for high-throughput DNA sequencing of complete human mitochondrial genomes

**DOI:** 10.1186/1471-2164-15-68

**Published:** 2014-01-25

**Authors:** Andrew C Clarke, Stefan Prost, Jo-Ann L Stanton, W Timothy J White, Matthew E Kaplan, Elizabeth A Matisoo-Smith

**Affiliations:** 1 Department of Anatomy, University of Otago, Dunedin, New Zealand; 2 Allan Wilson Centre for Molecular Ecology and Evolution, Dunedin, New Zealand; 3 Current address: School of Life Sciences, University of Warwick, Coventry, United Kingdom; 4 Department of Integrative Biology, University of California, Berkeley, California, USA; 5 Department of Mathematics and Statistics, University of Otago, Dunedin, New Zealand; 6 Human Origins Genotyping Laboratory, Arizona Research Laboratories, Division of Biotechnology, University of Arizona, Arizona, USA

**Keywords:** Human, Mitochondrial DNA, Next-generation sequencing, 454 sequencing, Long-range PCR, Bioinformatics

## Abstract

**Background:**

Next-generation DNA sequencing (NGS) technologies have made huge impacts in many fields of biological research, but especially in evolutionary biology. One area where NGS has shown potential is for high-throughput sequencing of complete mtDNA genomes (of humans and other animals). Despite the increasing use of NGS technologies and a better appreciation of their importance in answering biological questions, there remain significant obstacles to the successful implementation of NGS-based projects, especially for new users.

**Results:**

Here we present an ‘A to Z’ protocol for obtaining complete human mitochondrial (mtDNA) genomes – from DNA extraction to consensus sequence. Although designed for use on humans, this protocol could also be used to sequence small, organellar genomes from other species, and also nuclear loci. This protocol includes DNA extraction, PCR amplification, fragmentation of PCR products, barcoding of fragments, sequencing using the 454 GS FLX platform, and a complete bioinformatics pipeline (primer removal, reference-based mapping, output of coverage plots and SNP calling).

**Conclusions:**

All steps in this protocol are designed to be straightforward to implement, especially for researchers who are undertaking next-generation sequencing for the first time. The molecular steps are scalable to large numbers (hundreds) of individuals and all steps post-DNA extraction can be carried out in 96-well plate format. Also, the protocol has been assembled so that individual ‘modules’ can be swapped out to suit available resources.

## Background

Next-generation DNA sequencing (NGS) technologies have made huge impacts in many fields of biological research, but especially in evolutionary biology [1,2]. Concurrent with the increased use of NGS technologies has been an improved understanding of the amount and type of data required to answer certain types of evolutionary and population genetics questions. For example, where mitochondrial DNA (mtDNA) data are required, it is seen as increasingly necessary to obtain complete mitochondrial genomes. This is especially true in studies of humans, but for other animal species also [3]. The use of complete mtDNA genomes can help mitigate the reduced phylogenetic resolution, homoplasy and ascertainment bias that is otherwise encountered when using markers for known single nucleotide polymorphisms (SNPs) or shorter mtDNA sequences (e.g., control region) [4,5]. For human populations that are poorly studied, complete mtDNA genomes are even more important because there are often few known SNPs and, therefore, the relevant regions of the mitochondrial phylogenetic tree may be poorly resolved.

In addition to evolutionary applications, complete mtDNA genomes are also being sequenced to identify markers associated with mitochondrial disease, and the advent of NGS has seen the significant expansion of research in this area [6]. For example, NGS of mtDNA genomes is being used to clinically diagnose mitochondrial diseases in individuals with phenotypic evidence of mitochondrial oxidative phosphorylation disease [7]. The high sensitivity of NGS means it is also being used to discover diseases associated with low-level mitochondrial heteroplasmy that would be undetectable with conventional Sanger sequencing [8].

Despite the increasing use of NGS technologies and a better appreciation of their importance, there remain significant obstacles to the successful implementation of NGS-based projects. These challenges often relate to assembling the constituent components of a NGS sequencing protocol into a single workflow to suit a given study. NGS workflows are often complex, and necessarily span everything from the generation of suitable starting template, to various molecular biological steps, to the generation of the raw sequence data, and finally to the bioinformatic steps required to convert those data into a suitable format for downstream phylogenetic or population genetic analyses. New problems can arise when scaling up a protocol for use on tens or hundreds of individuals; protocols need to be robust and remain time efficient. The bioinformatics steps offer their own challenges because although many of the individual components/programs are available (e.g., for primer removal, mapping/assembly, and SNP calling) it is often difficult to get the outputs from one component into a format where they can be used as inputs for other components. Taken together, the challenges in assembling a complete NGS protocol represent a major source of inertia for researchers wanting to undertake NGS studies for the first time.

**Figure 1:**
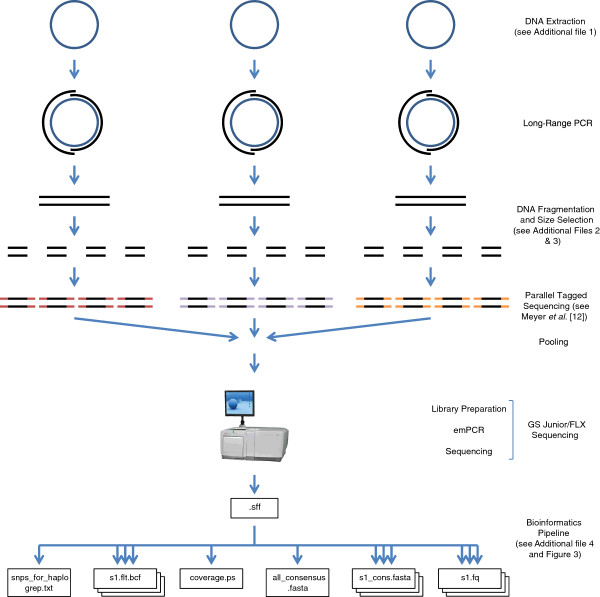
**Overview of the A to Z method for high-throughput DNA sequencing of complete human mitochondrial genomes.** DNA is collected using cheek swabs and then extracted using a phenol–chloroform method. Long-range PCR is used to amplify each mitochondrial genome in two overlapping amplicons. The two amplicons from each genome are then pooled and fragmented using NEBNext dsDNA Fragmentase. Barcoding of the fragments is then achieved using Parallel Tagged Sequencing (PTS) [12]. Barcoded fragments are then pooled for library preparation, emulsion PCR (emPCR) and pyrosequencing on the 454 GS FLX platform. Using a number of bioinformatics tools, the resulting sequence data are de-multiplexed and barcodes and primers are removed. Reference-based mapping (to a circular reference) is carried out, followed by the output of coverage plots, consensus sequences and SNP calling for each individual.

Here we present an ‘A to Z’ protocol for obtaining complete human mitochondrial (mtDNA) genomes – from DNA extraction to consensus sequence. Although designed for use on humans, this protocol could also – with minor modifications – be used to sequence small, organellar genomes from other species, and also nuclear loci. An overview of the A to Z method is presented in Figure 1.

All steps in this protocol are designed to be straightforward to implement; although the particular combination of steps is novel, the reagents and recommended equipment are widely available, and the bioinformatics is easily performed by non-experts (and can be modified where necessary). The molecular steps are scalable to large numbers (hundreds) of individuals and all steps post-DNA extraction can be carried out in 96-well plate format (throughput is often a limitation in other protocols). Also, the protocol has been assembled so that individual ‘modules’ can be swapped out to suit different scientific questions, facilities, skill sets and budgets. Some of these alternatives are described in the protocol.

It should be noted that the protocol here is largely kit-based, with speed, efficiency and throughput the main priorities. Labs with limited consumables budgets may wish to investigate non-kit-based alternatives for some of the more expensive steps. Non-kit-based methods may, however, decrease reliability and increase labour costs through additional time spent preparing reagents, etc. The decision of whether to use a kit or a non-kit alternative may also depend on access to the equipment required in either case, and equipment requirements should be thoroughly investigated beforehand.

Both consumables and labour costs vary dramatically across countries, and therefore we have not included a cost analysis for this protocol because it would not be broadly applicable. Instead, it is recommended that researchers create a budget before using this protocol, where the costs of consumables are balanced against labour costs, the technical expertise required for different methods, the number of samples to be processed and the deadlines for the project.

Although initial sample preparation is still largely carried out by researchers ‘in-house’, it is increasingly common to take advantage of the significant cost savings associated with out-sourcing NGS to an external provider. As such, researchers using the early steps of the protocol may wish to investigate out-sourcing the wet lab stages from library prep onwards. The decision of whether or not to use an external sequencing provider should also form part of the project plan.

## Methods

### Sample collection and DNA extraction

DNA was collected from participants using a buccal swab. Two swabs were obtained for each participant (one for immediate use, and one as a back-up). All samples were obtained with informed consent (University of Auckland Human Participants Ethics Committee (UAHPEC), Ref. 2008/203). DNA was extracted from the cheek swabs using a phenol–chloroform method (see Additional file 1 for the full extraction protocol). Briefly, DNA samples were digested overnight with proteinase K and then extracted with phenol, chloroform and isoamyl alcohol. DNA was precipitated with isopropanol, washed with ethanol and eluted in Low TE (10 mM Tris (pH 8.0), 0.1 mM EDTA (pH 8.0)). DNA was visualised by running 5 μL aliquots of each extraction on a 1% (w/v) agarose gel, and successful DNA extractions were confirmed by the presence of a band of high molecular weight DNA. DNA samples were quantified using a PicoGreen quantification assay.

### Long-range PCR amplification of complete mtDNA genomes

Long-range PCR (LR-PCR) is an efficient method for generating template for sequencing, especially in well-characterized taxa where LR-PCR primers can be designed easily (e.g., mammals and birds). In less well studied lineages where primer design can be problematic, Rolling Circle Amplification (RCA) has shown to be an effective alternative to LR-PCR for generating template for NGS [9]. Even when LR-PCR is possible, some modification of the protocol may be necessary, such as for species that have AT-rich mtDNA genomes [10].

The complete human mt genome was amplified from each individual by generating two overlapping long-range PCR products of 8511 bp (HumLR_1) and 8735 bp (HumLR_2) (Table 1). The LR-PCR primers were designed using Primer3Plus (http://www.bioinformatics.nl/cgi-bin/primer3plus/primer3plus.cgi), and with no, or very weak, predicted secondary structures. The primer-binding sites were positioned to be conserved across the 127 complete mt genomes in the dataset of Pierson et al. [11].

**Table 1:** Long-range PCR primers for amplifying the complete human mitochondrial genome

**Primer name**	**Sequence (5′–3′)**	**Length (nt)**	**5′–3′ binding position in revised Cambridge Reference Sequence (rCRS)**	**Expected product size (bp)**
HumLR_1F	ACGGGAAACAGCAGTGATTAAC	22	807–828	8511
HumLR_1R	CTAGTATGAGGAGCGTTATGGAGTG	25	9342–9318	
HumLR_2F	GTACGCCTAACCGCTAACATTACT	24	8998–9021	8735
HumLR_2R	GTTTTAAGCTGTGGCTCGTAGTG	23	1163–1141	

LR-PCR products were amplified using the Expand Long Range dNTPack (Roche). Individual reactions contained 1× Expand Long Range Buffer (with 2.5 mM MgCl_2_), 0.5 mM of each dNTP, 0.3 μM forward primer, 0.3 μM reverse primer, 3% (v/v) dimethyl sulfoxide (DMSO), 2 U enzyme mix, and 1.5 μL (50–500 ng) genomic DNA in a total volume of 30 μL. Thermal cycling conditions were: initial denaturation at 92°C for 2 min; followed by 10 cycles of denaturation at 92°C for 10 s, annealing at 55°C for 15 s, and extension at 68°C for 8 min 30 s; followed by 25 cycles of denaturation at 92°C for 10 s, annealing at 55°C for 15 s, and extension at 68°C for 8 min 30 s, with the extension time increasing 20 s/cycle for each subsequent cycle; followed by final extension at 68°C for 7 min; followed by a hold at 10°C.

PCR products were visualised by electrophoresis of a 2 μL aliquot of the PCR on a 1% (w/v) agarose gel. Successful PCRs were represented by a bright band (6–15 μg DNA) of the expected size.

### Purification and quantification of LR-PCR products

LR-PCR products were purified using the AMPure XP Kit (Beckman Coulter) and solid-phase reversible immobilization (SPRI) technology. Purifications were carried out using 1.8 volumes (i.e., 1.8 × the sample volume) of AMPure XP and exactly as described in Steps 5–8 of Meyer et al. [12]. Purified DNA was eluted in 25 μL of 10 mM Tris (pH 8.0). The significant advantage of the SPRI technology is that, using a multi-channel pipette, an entire 96-well plate can be purified in less than 2 hours. Smaller numbers of samples can be purified using individual columns (e.g., QIAquick PCR Purification Kit (Qiagen)) but this is very time-consuming and expensive with large numbers of samples.

A PicoGreen quantification assay was used to accurately quantify the purified LR-PCR products prior to fragmentation. To ensure the concentration values of the samples fell within the linear section of the standard curve, it was necessary to dilute an aliquot of the purified samples 20-fold, although the exact dilution required will depend on the quantification setup.

### Fragmentation of PCR products using NEBNext dsDNA fragmentase

For each individual, the two LR-PCR products (HumLR_1 and _2) were pooled in equimolar ratios (493.5 ng and 506.5 ng respectively) to yield a total of 1 μg DNA for fragmentation. Next, the pooled DNA was fragmented using the NEBNext dsDNA Fragmentase according to the manufacturer’s instructions and Additional file 2. Briefly, dsDNA Fragmentase generates dsDNA breaks in a time-dependent manner, producing 100–800 bp fragments, depending on incubation time. Note that the optimum incubation time must be determined empirically as described in Additional file 2, although we found it to be between 10 and 18 minutes.

Alternatively, sonication may be used instead of Fragmentase. We have used the Bioruptor® Pico sonication system, which has provided successful fragmentation following the manufacturer’s instructions and a 15 s/90 s on/off cycle time for 7–8 cycles.

### Purification and quantification of fragmentase reactions

Fragmentase reactions were purified using the Polyethylene Glycol–Bead (PEG–Bead) Solution described in Additional file 3. Briefly, the beads are isolated from AMPure XP solution and resuspended in a 10–30% PEG solution, with the percentage determining the size cut-off below which fragments are removed. Purifications were carried out using 1.8 volumes (i.e., 1.8 × the sample volume) of PEG–Bead Solution and exactly as described in Steps 5–8 of Meyer et al. [12]. Purified DNA was eluted in 20 μL of 10 mM Tris.

**Figure 2:**
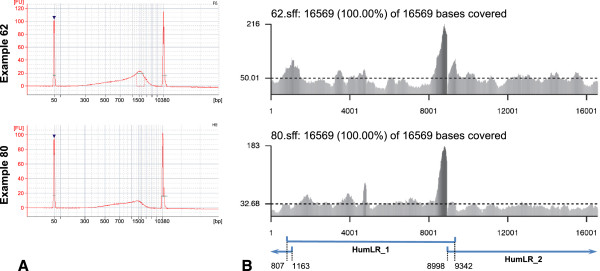
**Example fragmentation profiles and coverage plots.** Example fragmentation profiles and coverage plots are shown for two mitochondrial genomes (examples 62 and 80). **A**. These Bioanalyzer profiles (from a DNA 7500 chip) show pooled long-range PCR products after digestion with NEBNext dsDNA Fragmentase (10 min at 37°C). The *x*-axis shows the inferred size of the DNA fragments based on the two internal markers of known size (the peaks at 50 and 10,380 bp). The *y*-axis shows the amount of DNA present based on fluorescence units. Both example digestion profiles show fragments between distributed between ~300 bp and ~5 kb in length, with the distribution skewed towards smaller fragments. These profiles show fragments in the ideal size range for 454 sequencing. The difference in yields between the two samples is probably due to different recovery efficiencies in the preceding AMPure XP purification step. Screen captures are taken from the 2100 Expert software (Agilent). **B**. These coverage plots for two mitochondrial genomes were generated using the software described in this paper. The *x*-axis shows the nucleotide positions based on the revised Cambridge Reference Sequence (rCRS). The *y*-axis shows coverage depth. The horizontal dashed line indicates mean coverage for that genome. On the left of each heading line is the individual name (e.g. 62.sff or 80.sff); the following number (here, 16569) is the number of positions that were covered by at least 1 read, and the final number (here, also 16569) is the length of the reference sequence. Note the large peak from 8,000–9,000 bp, which is discussed in the main text. The blue lines represent the corresponding long-range PCR products and the associated numbers the positions of the ends of those products (see Table 1). The data used to generated these coverage plots is available in Additional file 6.

A subset of the purified Fragmentase reactions was run on the Bioanalyzer 2100 using a DNA 7500 chip to ensure that fragments were within the desired size range (400–1000 bp). Typical Bioanalyzer fragment profiles are shown in Figure 2A. A PicoGreen quantification assay was used to accurately quantify the purified Fragmentase reaction products prior to barcoding.

### Barcoding for parallel tagged sequencing

Barcoding and pooling was carried out exactly as described in Meyer et al. [12], except that the AMPure XP Kit was used in place of the AMPure Kit.

### Sample library construction and sequencing

Sequencing using GS FLX or GS Junior (454 Life Sciences/Roche, Germany) offers a complete system for preparing sequencing libraries and generating sequence data. In concert with the instrument, kits were used for constructing libraries, carrying out emPCR and sequencing the samples. Most of the components required to undertake these processes are supplied in these kits, the exceptions being a system to accurately quantify DNA and reagents for performing essential quality control on sequencing libraries. Below we briefly summarise the GS Junior sequencing process.

Fragmented and barcode-tagged samples must be accurately quantified before commencing library construction. We recommend using a fluorometric method, for example, with PicoGreen or the Qubit system (Cat Number Q32866, Life Technologies, USA). Fragmented, tagged samples from different individuals are mixed in equal amounts to form a single pool of DNA molecules. This pooled sample is used to construct the sequencing library. Adding equal amounts of DNA from each individual ensures equal representation of these sequences in the final data output. In the example described here (whole human mt genomes) the samples are of equal length. If samples of different length are pooled for library construction then the mass of DNA used for each sample should be adjusted accordingly to ensure coverage levels are the same across all samples (see ‘Fragmentation of PCR products using NEBNext dsDNA fragmentase’ above). We have successfully constructed libraries from pooled DNA samples ranging from 80 ng to 750 ng in total.

A single Rapid DNA library was constructed from each pooled sample using the FLX Titanium Rapid Library Kit (454 Life Sciences/Roche). Library construction results in the ligation of specific adaptors to the ends of the DNA molecules. Following library construction, DNA fragments less than 500 bp in length, including unligated adaptors, were removed from the sample using the AMPure XP Kit (Beckman Coulter). This was achieved by first isolating the DNA-bead mixture on a SPRIPlate and then discarding the derived buffer as per the manufacturer’s protocol. The AMPure XP beads were then washed in Size Solution (supplied with the Rapid Library Kit, 454 Life Sciences/Roche, Germany) to remove short DNA fragments and any buffer components from previous reactions. Two washes using 70% (v/v) ethanol were then carried out according to the AMPure XP protocol. The bead pellet was air-dried completely and the size-selected sequencing library eluted by resuspending the pellet in 53 μL of TE buffer (10 mM Tris (pH 8.0), 1 mM EDTA (ph 8.0)). The sequencing library was transferred to a clean tube by drawing down the beads with the magnet prior to transfer.

Library quality was determined in two ways. First, one of the sequencing adaptors is supplied pre-labelled with fluoroscein isothiocyanate (FITC), allowing a fluorescent plate reader to be used to determine the concentration of the library in molecules/μL (based on a standard curve). Standards are supplied with the GS FLX Titanium Rapid Library Kit (454 Life Sciences/Roche). In our experience, these libraries yielded between 4.19 × 10^8^ and 5.5 × 10^8^ molecules/μL. Second, the size distribution of the sequencing library is determined from a 1 μL aliquot run on a High Sensitivity DNA Chip (Agilent Technologies) on the Bioanalyzer instrument (Agilent Technologies). Sequencing library DNA fragment size distribution should be between 350 bp and 2000 bp with a peak distribution around 700 bp. Libraries with fragment sizes significantly outside of this range should not be used; this indicates that Fragmentase reaction conditions should be reoptimised for DNA preparation.

Preparation of the library for sequencing starts with emulsion PCR (emPCR) [13], which was carried out using the GS Junior Titanium emPCR Kit (Lib-L) (454 Life Sciences/Roche). This process begins with binding a single DNA molecule from the library to a single Sequence Capture Bead. The amount of DNA added is critically important: if too much DNA is added, the beads will bind multiple DNA molecules resulting in mixed sequence on each bead and as a result will be unreadable; if not enough DNA is added, the emPCR will not deliver enough Sequence Capture Beads for efficient sequencing. For this project, a ratio of two DNA molecules per bead was used. The volume of library to add to an aliquot of Sequence Capture Beads is calculated using the equation (1):

(1) $$\mathrm{\mu L}\;\mathrm{of}\;\mathrm{library}=\frac{\mathrm{molecules}\;\mathrm{per}\;\mathrm{bead}\times 10\;\mathrm{million}\;\mathrm{beads}}{\mathrm{library}\;\mathrm{concentration}\;\left(\mathrm{molecules}/\mathrm{\mu L}\right)}$$

The steps from emulsion formation to biotin–streptavidin-enrichment were carried out according to the manufacturer’s protocols. Following the enrichment process, approximately 500,000 Sequence Capture Beads should remain. Fewer than 500,000 Sequence Capture Beads will be insufficient for a sequencing run. More than 1.5 million beads remaining after enrichment indicates that there are too many beads coated with multiple sequences. These beads should not be used, as the sequences they hold cannot be resolved and they will be discarded from the final data set.

Sequencing was performed using the GS Junior and a PTP Kit and Sequencing Kit (454 Life Sciences/Roche, Germany). This method of pyrophosphate-based sequencing is described elsewhere [14,15]. Each run took 10 hours to complete. Control beads seeded onto the PicoTiterPlate (PTP) at the time of loading independently indicated both the chemical and instrument performance of each sequencing run. The output from the sequencing run is a computer file (.sff), containing quality scores and raw data for each sequence generated from the run. Only those sequences that pass five quality filters are present in the final data set. This ensures only high quality sequence reads progress into the analysis phase of the project. The final output from the GS Junior typically yielded between 60,000 and 100,000 quality sequence reads with an average length of between 350 and 450 bp.

### Computational raw data processing

**Figure 3:**
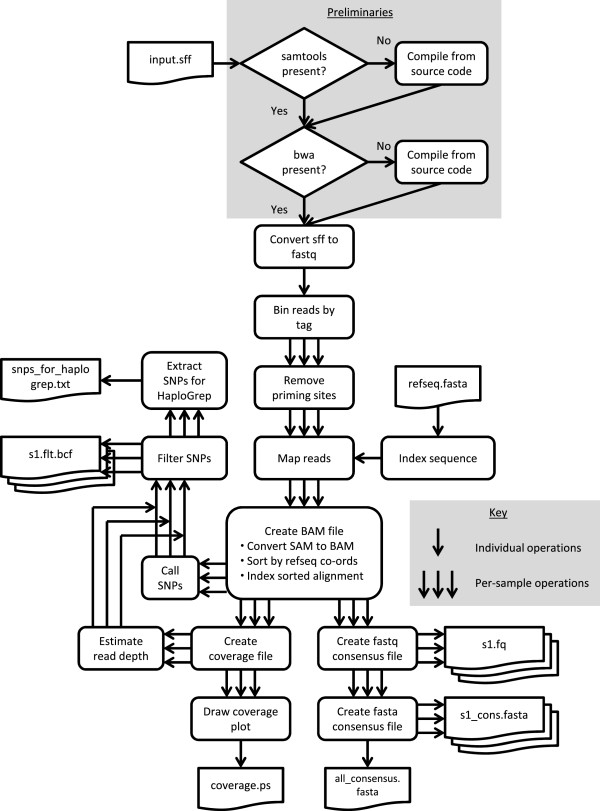
**Steps in the bioinformatics pipeline.** The pipeline automatically converts a single .sff input file from a 454 run containing the raw sequencing data for one or more individuals into a variety of useful output files, notably including per-individual consensus sequence and coverage plot files. To simplify usage it begins by detecting whether the necessary C programs have been installed, and automatically compiling bundled versions if not, before proceeding to the bioinformatics steps.

In the next step the raw sequencing data are processed for use in downstream analysis (see Figure 3 for an overview). Here we present an easy-to-use bash-script-based pipeline that allows the user to automatically process sequencing files for single or multiple individuals. The presented pipeline runs on all UNIX-based operating systems. The step-by-step protocol is provided in Additional file 4 and the associated scripts in Additional file 5. Additional file 6 contains two example .sff files that can be used to perform test runs of the scripts. The presented pipeline consists of freely available standard tools for read mapping and post-processing, such as BWA [16], SAMtools [17] and our own scripts, which complement these tools. All incorporated scripts run either on Python or Perl, which should be pre-installed on UNIX operating systems. It can be used to map and process sequencing reads from different data sources, such as evolutionary genetics, medical research or even short, damaged ancient DNA reads (see [18]).

The presented pipeline first sorts individual reads according to their barcodes (for multiplexed libraries), then removes PCR priming sites, performs reference-based mapping and finally genotype and variant calling. Different data quality and quantity statistics are included. These steps are discussed in more detail below.

### De-multiplexing, removal of barcodes and priming sites and reference-based read mapping

In the first data processing step the .sff file is converted into a fastq file using sff_extract_0_2_13 (http://bioinf.comav.upv.es/_downloads/sff_extract_0_2_13). The fastq format is similar to the commonly used fasta format, but also stores data quality scores in addition to the sequence information. The reads are then separated into per-individual fastq files according to their barcode using nuntag.pl (Additional files 4 and 5). Nuntag is based on untag (https://bioinf.eva.mpg.de/pts/), but is coded in Perl and should thus be much easier to run than untag, which requires a Haskell compiler and various additional libraries to be installed. The source code for untag is included in Additional file 5 and, once it has been correctly installed, switching the pipeline to use it is straightforward.

In the next step, priming sites have to be removed from the reads because the primer sequence can vary from the priming site and thus might lead to calling false sequence variation. The tagcleaner software [19] (http://tagcleaner.sourceforge.net/) was used to remove the long-range priming sites. This software tool looks for the specific sequences within a specified distance to the 5′ and the 3′ end of the reads. To account for mismatches and partial primer sites present, the last five nucleotides of the respective primers were used. The trim_within regions was specified as 26 for HumLR_1F and HumLR_1R and 25 for HumLR_2F and HumLR_2R, respectively. Alternatively, the freely available software tool AdapterRemoval [20] can be used.

In the next step the cleaned reads are aligned using reference-based mapping. To do so, the pipeline applies the Burrows–Wheeler Alignment Tool (BWA) with the *bwasw* algorithm, which uses heuristic Smith–Waterman-like alignment to find high-scoring local hits [16]. This approach is very powerful when applied to long read data with a high error rate, but can be slower and less accurate for short low-error mappings [21]. The revised Cambridge Reference Sequence (rCRS [22]) was used as a reference for the mapping. Alternatively, other sequences can be used. For comparing called SNPs across datasets, the same reference is required for each. In some cases, the rCRS might differ substantially from the consensus sequence of the processed reads. In this case a second mapping against a reference for the inferred haplotype might lead to more reads being mapped.

### Downstream variant and haplotype calling

The resulting sam file is then processed with the software SAMtools [17] to call the consensus sequence and variants such as SNPs. It should be noted that SAMtools 0.1.18 treats N’s in the reference as A’s when calling the consensus. Furthermore, wherever a region of the reference is covered by a single or multiple gaps in the reads, the program will call the nucleotide(s) of the reference instead of the gap. Thus, it is recommended that suspicious SNPs or regions in the original mapping are checked.

In the following step, the filtered SNPs output from bcftools (part of the SAMtools software package) are transformed into an input file for the haplogroup-assigning tool HaploGrep (http://haplogrep.uibk.ac.at/) using a Perl script (see Additional file 4). The haplotypes can then be called online (or locally) using HaploGrep.

It should be noted that the current setup does not allow for calling of indels. Indels are insertions or deletions of point mutations. In recent years indels in mitochondrial DNA and mitochondrial DNA analysis in general have gained wide interest in genetic medicine [23-25]. However, data produced on the 454 platform shows an increased rate of false-positive SNPs [26-28], due to problems in calling the correct number of nucleotides in polynucleotide stretches because of signal-to-noise threshold issues. This limitation might be overcome by deeper sequencing (higher coverage of the position in question). However, studies have shown that a higher coverage is not sufficient to overcome this effect if homopolymeric nucleotide stretches are longer than 10 nucleotides [26,29]. Studies in which indels are particularly important, such as on human diseases [24,25], might need to adapt the approach by deeper sequencing and allowing SAMtools to call indels (see online supplementary bioinformatics protocol) or by avoiding using 454 altogether. It is recommended that indels are called using technologies with low indel error rates, such as Illumina.

Heteroplasmy (the presence of more than one mitochondrial haplotype per cell) is a common phenomenon in human mitochondrial DNA. Thus, by default the pipeline includes ambiguity codes in the consensus sequence. However, it should be noted that the downstream haplotype assignment using HaploGrep does not support heteroplasmic sites. Therefore, the major nucleotide has to be determined by eye prior to using Haplogrep. In cases of heteroplasmic length polymorphisms a *de novo* approach might be more appropriate than reference-based mapping (see below).

### Read quality

The data quality of the mapped reads can be checked using the freely available software tool FastQC (http://www.bioinformatics.babraham.ac.uk/projects/fastqc/). FastQC can be used to infer sequence quality scores, GC content, read lengths distribution and to identify overrepresented sequences. Base-calling algorithms, such as Pyrobayes [27] for 454 data, produce per-base quality scores by analysis of incorporation signals, so-called Phred scores [30,31]. A Phred score of 20, for example, means that there is a 1 in 100 chance that the read is incorrectly mapped [31]. The distribution of Phred scores can easily be assessed using FastQC. The software is further able to assess quality values such as read length, sequence GC content, etc. If the read quality is low, reads can be trimmed e.g. with the freely available software tool trimmomatic [32].

### Coverage plots

Coverage plots showing the number of reads overlapping each position in the reference genome are useful for quickly assessing mapping quality (see Figure 2B). The presented pipeline (Additional file 5 and online supplementary bioinformatics protocol: Additional file 4) automatically produces a coverage plot for each sample, which shows coverage level versus reference position as a greyscale bar graph and the average coverage level as a dashed line. Plots are broken across lines and pages as necessary; the default scale factor fits up to 20,000 nucleotides per row and 5 rows per page, which is convenient for examining multiple mitochondrial genomes, but these settings can be adjusted by the user. The plots are in high-resolution (vector) PostScript format and can be viewed or printed using the freely available GhostView program (http://pages.cs.wisc.edu/~ghost/). A healthy coverage plot shows near-average coverage across the genome.

All coverage plots we have generated show a peak of high coverage between 8,000–9,000 bp, which roughly corresponds to the location of *atp8*. At the time of writing, we do not have an explanation for the high number of reads at this location, but we have ruled out low GC content (which would cause the dsDNA Fragmentase to cut more frequently) and it does not appear to be associated with the end of the long-range fragments, as no equivalent peak appears near the overlap of HumLR_1 and _2 between positions 807 and 1163. We have also ruled out any artefacts associated with the bioinformatics pipeline. The consistent appearance of this peak across all individuals suggest that it is a product of the nucleotide order in this region (that, for example, may affect secondary folding and ultimately the number of reads derived). This high coverage region has no effects on the consensus sequence obtained.

### Important considerations for alignment and assembly

A critical decision is the choice of the appropriate strategy for alignment and assembly of the sequencing reads. In general, two approaches can be used to obtain a consensus sequence or an assembly to call variants: “reference-based mapping” and “*de novo* assembly”. If a high-quality reference sequence is available, as in the case of the human mitochondrial genome, the sequencing reads can be mapped against this reference. Reference-based mapping has some advantages over *de novo* assembly. Since the reads are mapped against a reference, reads can be assembled even if regions in between are poorly or not at all covered (even without paired-end sequencing libraries). This allows consensus sequences to be generated even in the presence of missing data. Furthermore, contamination or sequencing artefacts are usually filtered because they are unlikely to align to the reference.

Reference-based mapping is commonly used in human genetic studies that are based upon mitochondrial genomes. A multitude of freely available software tools for reference-based mapping are available, including the commonly used software tools BWA [16] and Bowtie 2 [33]. Available mitochondrial genome sequences such as the revised Cambridge Reference Sequence (rCRS) can be used for the mapping. However, due to the mapping algorithms, problems can occur in cases such as duplication or deletion of genomic regions. For example, a commonly found motif in mitochondrial genomes obtained from the Pacific region is the deletion of a 9 bp (CCCCCTCTA) repetitive sequence, located between the cytochrome oxidase II (*COII*) and lysine tRNA (*tRNA*^*Lys*^) genes. This motif is commonly present in two copies in Europeans and only one in the Pacific or Asia [34]. Reference-based mapping of reads from an individual having only one copy against a reference containing two copies (such as the rCRS) can lead to false consensus calling. This phenomenon is due to the possibility of aligning this motif either to the first or the second copy in the reference. If different reads are aligned to different copies, the consensus will call both copies (see Figure S1 in Additional file 4). This problem can be overcome either by applying realignment tools (such as GATK [35]) or *de novo* assembly. Unfortunately GATK cannot handle 454 data and was thus not included in the pipeline. For known deletion or insertion of repeats, the excessive copy in the reference can be substituted by “-”, which allows for mapping of the same number of or fewer copies.

*De novo* assembly is a powerful approach to align reads if no high-quality reference sequence or sequence of a closely related taxon is present. Different free software tools are available to perform *de novo* assembly, such as Velvet [36], MIRA 3 [37] or Newbler (454 software; http://my454.com/products/analysis-software/index.asp). For a detailed review on available methods see [38,39]. *De novo* assembly is based upon the redundancy of short-read sequencing and the resulting possibility to find overlapping sequencing reads. This approach strongly benefits from the availability of longer reads (such as from 454 data) or the sheer number of data reads provided by next-generation sequencing platforms (such as the Illumina HiSeq, etc.). The advantages of *de novo* assembly are that it is independent of any reference sequence and that it can be used to detect variants on a population level (see discussion of repetitive sequence mapping above). Disadvantages include substantially higher computational requirements and problems resolving contigs (sequence fragments inferred from clusters of overlapping reads) into the correct linear order. Although not implemented in the presented protocol, software tools such as MIRA 3 [37] (http://sourceforge.net/projects/mira-assembler/) or the standard 454 *de novo* assembler Newbler 2.5 (http://my454.com/products/analysis-software/index.asp) can also be easily implemented in the pipeline if desired.

## Results and discussion

### DNA extraction

DNA extractions typically yielded 1–50 μg of high molecular weight total genomic DNA (that probably also includes a large proportion of microbial DNA). This DNA was suitable for routinely amplifying long-range PCR products (~9 kb). In addition, DNA extracted using this method was found to be stable at 4°C for at least 2 months, avoiding the need to repeatedly freeze–thaw the DNA samples.

### Long-range PCR amplification of complete mtDNA genomes

The long-range primers described in Table 1 have been used to amplify mt genomes from phylogenetically diverse individuals (including from haplogroups B, L3*, P, Q, H, W and T), but it is possible that mutations in the primer-binding sites for some haplogroups may interfere with amplification. In these cases, it might be necessary to redesign the primers for different primer-binding sites, or to include degenerate bases.

The LR-PCR proved to be highly reliable, with > 95% of individuals yielding both LR-PCR products on the first amplification attempt. When the PCR failed, it was usually for both products of an individual, suggesting a problem with the DNA, rather than with the PCR itself. In these cases, if a second PCR attempt also failed then that individual was re-extracted using the back-up buccal swab.

The concentration of the undiluted purified LR-PCR products was typically 200–500 ng/μL.

### Fragmentation of PCR Products

Although it is necessary to optimise the dsDNA Fragmentase reactions for a given template, this proved to be an efficient method for fragmenting the long-range PCR products of a large number of individuals, and produced consistent results. Although mechanical shearing or sonication methods (e.g., Covaris and Bioruptor) may be suitable for small numbers of samples, enzymatic fragmentation allows higher throughput.

### Bioinformatics

The presented pipeline is an easy-to-use Unix shell script that runs a series of programs (such as TaqCleaner, BWA, SAMtools) to transform a set of raw-read input files into a variety of useful output files. In addition to applying existing freely available software tools, new scripts have also been developed, e.g. to produce coverage plots, which show the number of reads overlapping each position in the reference genome for easy quality assessment, and to convert SAMtools output files into HaploGrep input files for convenient haplotype calling. The pipeline has been set up to work for reads from the 454 sequencing platform (Roche), but it can easily be adjusted to be used for different platforms such as Illumina Miseq, Hiseq or IonTorrent (Life Technologies). Due to its modular organization, it is straightforward to change different parts of the data processing. Recently, Wilm et al. [40] presented LoFreq, a freely available variant calling tool (http://sourceforge.net/projects/lofreq/), that has similar precision to SAMtools [17] but shows a higher sensitivity in calling rare variants. Alternative tools, such as LoFreq, can easily be incorporated into the processing. Our pipeline can process reads for hundreds of sequences in a very short amount of time (depending on the number of reads this is typically only a few minutes on a desktop computer for mtDNA data). The performance is strongly dependent on the different tools used in the processing. For detailed discussions on the performance for different steps such as variant calling please see the publications for the respective tools.

## Conclusions

Here we present a protocol for sequencing complete human mitochondrial genomes. This protocol could, however, be used to sequence mitochondrial genomes from other species, and also nuclear loci of a similar length. Our aim is for this protocol to help researchers who are new to next-generation sequencing make full use of this technology. The benefits of this protocol include that it is straightforward to implement, and that the molecular steps are scalable to large numbers of individuals. The bioinformatics modules are designed to be reasonably easy to use for researchers new to command line-based inputs. Conscious of the different questions, facilities, skill sets and budgets available across research groups, we have assembled the protocol so that individual ‘modules’ can be changed to suit a particular project.

## Competing interests

The authors declare that they have no competing interests.

## Authors’ contributions

ACC developed the molecular biology protocols, drafted the manuscript and oversaw the integration of protocol components into a single workflow. SP and WTJW developed the bioinformatics protocols and drafted the manuscript. JALS carried out the library construction and sequencing, advised on the molecular biology protocols and drafted the manuscript. MEK developed the DNA extraction protocol. EAMS contributed to the project design, provided overall guidance to the project and revised the manuscript. Members of the Genographic Consortium assisted with the project design and revised the manuscript. All authors read and approved the final manuscript.

## Supplementary Material

Additional file 1 **Phenol–chloroform DNA extraction protocol.** PDF file of the phenol–chloroform protocol for isolating human genomic DNA from buccal (cheek) swabs. Click here for file

Additional file 2 **Fragmentase digestion protocol.** PDF file of the protocol for fragmenting LR-PCR products with NEBNext® dsDNA Fragmentase™. Click here for file

Additional file 3 **Size selection protocol.** PDF file of the protocol for DNA size selection using AMPure XP-derived PEG–Bead solution. Click here for file

Additional file 4 **Bioinformatics pipeline.** PDF file of the bioinformatics protocol. Click here for file

Additional file 5 **Bioinformatics pipeline.** tar.gz file (compressed tar archive) of the bioinformatics scripts. Click here for file

Additional file 6 **Example .sff files.** tar.gz file (compressed tar archive) of two example .sff files. Click here for file
