# Supplementary material for: From cheek swabs to consensus sequences: an A to Z protocol for high-throughput DNA sequencing of complete human mitochondrial genomes
Source: BMC Genomics. 2014 Jan 25;15:68. doi: 10.1186/1471-2164-15-68 (PMC3922791; doi:10.1186/1471-2164-15-68)
Supplement: Additional file 1 — Phenol–chloroform DNA extraction protocol. PDF file of the phenol–chloroform protocol for isolating human genomic DNA from buccal (cheek) swabs. [file 1471-2164-15-68-S1.pdf]

PROTOCOL

Isolation of Human Genomic DNA from Buccal (Cheek) Swabs

If using this protocol, please cite:

Clarke AC, Prost S, Stanton J-AL, White WTJ, Kaplan ME, Matisoo-Smith EA, The Genographic Consortium: **From cheek swabs to consensus sequences: an A to Z protocol for high-throughput DNA sequencing of complete human mitochondrial genomes.** *BMC Genomics* 2014, **15:**68.

For updates to this protocol, please visit http://www.clarkeresearch.org/AtoZ.html

Materials

Where a supplier is mentioned, this represents the supplier we have used; other suppliers' products may produce comparable results.

Reagents

All reagents should be an appropriate molecular biology grade.

- Swabs ("Omni Swabs" available from Whatman, cat. no. WB100035)
- 2.0 mL screw-cap tubes (available from Sarstedt, cat. no. 72.694.106)
- Lysis buffer (50 mM Tris (pH 8.0), 50 mM EDTA, 25 mM sucrose, 100 mM NaCl, 1% SDS)
- Proteinase K (20 mg/mL)
- Low TE (10 mM Tris (pH 8.0), 0.1 mM EDTA (pH 8.0)
- 3 M NaOAc (pH 5.2) (available from Sigma, cat. no. S7899)
- 70% EtOH (ethanol)
- 95–100% EtOH (ethanol)
- Phenol equilibrated with Tris, pH 8.0 (available from Sigma, cat. no. P4557)
- $CHCl_3$:IAA (24 parts chloroform : 1 part isoamyl alcohol) (available pre-mixed from Sigma, cat. no. 25666)

Procedure

Swab collection

1. Swab/scrape the inside of the cheek firmly for approximately 30 s.
2. Eject swab into a 2.0 mL screw-cap tube filled with 750 µL lysis buffer. Seal tube and store at room temperature. In this form, swabs will keep for years.

Day 1 – Proteinase K digestion

3. Add 12.5 µL of 20 mg mL−1 proteinase K to the tube containing the swab.
4. Incubate the sample at 55°C with rotation overnight (e.g., in a hybridisation oven).

Day 2 – DNA extraction

! CAUTION Ensure standard protocols are followed when dealing with phenol and chloroform, including carrying out steps 6, 9 and 11 in a fume hood.

5. Briefly spin down tube.
6. Add one volume (750 µL) of saturated, pH adjusted (pH 8.0) phenol and 1/10 volume (75 µL) of CHCl3:IAA (24:1). Mix by gentle inversion.
7. Centrifuge for 5 min at maximum speed to separate the phases.
8. Slowly pipette the aqueous phase (usually 550 µL) into a new 1.7 mL tube. Avoid the interface and transfer only clean aqueous solution.
9. Add an equal volume (550 µL) of CHCl3:IAA (24:1). Mix by gentle inversion.
10. Centrifuge 5 min at maximum speed to separate the phases.
11. Slowly pipette the aqueous phase (usually 400 µL) into a new tube. Avoid the interface and transfer only clean aqueous solution.
12. Add 1 volume (400 µL) of isopropanol and 1/10 volume (40 µL) of 3M NaOAc. Mix by gentle inversion.
13. Incubate on ice for 1–2 hours to precipitate the DNA.

    ▲ **CRITICAL STEP** Incubating for periods longer than two hours (e.g., overnight) may cause salt to co-precipitate with the DNA.
14. Pellet the DNA by centrifugation at maximum speed at 4°C for 30 min.

    Note: Orientate the tube so that the hinge is facing the outside of the centrifuge. This will help in avoiding the (sometimes invisible) pellet when the alcohol is being removed.
15. Remove the liquid using a pipette, taking care not to disturb the pellet.
16. Wash the pellet with 900 µL 70% EtOH. Mix by gentle inversion.

    Note: Using 900 µL of EtOH (rather than 1000 µL) will make it easier to remove all the EtOH with a 1000 µL pipette.

17. Pellet the DNA by centrifugation at maximum speed at 4°C for 30 min.
18. Remove the liquid using a pipette, taking care not to disturb the pellet.
19. Repeat steps 12–14 once for a total of two washes with 70% EtOH.
20. Wash the pellet with 900 µL 95–100% EtOH. Mix by gentle inversion.
21. Pellet the DNA by centrifugation at maximum speed at 4°C for 30 min.
22. Remove the liquid using a pipette, taking care not to disturb the pellet.

    ▲ CRITICAL STEP The pellet will be very loose at this point.
23. Leave the lid open and air-dry the samples overnight to ensure they are dry. Tubes should be covered (e.g., by placing them in a drawer).

Day 3 – Dissolving of DNA

24. Visually inspect the samples to ensure there is no residual ethanol.
25. Resuspend the DNA in 50 µL Low TE (pH 8.0). Incubate the DNA in the fridge (+4°C) overnight.

    ▲ CRITICAL STEP You can flick the tube several times to help the DNA dissolve but DO NOT pipette the DNA up and down as this will shear it.
26. DNA can be quantified by electrophoresis on a 1.0% agarose gel or by performing a PicoGreen assay, etc. Yields vary from 1–50 µg.
