# Supplementary material for: From cheek swabs to consensus sequences: an A to Z protocol for high-throughput DNA sequencing of complete human mitochondrial genomes
Source: BMC Genomics. 2014 Jan 25;15:68. doi: 10.1186/1471-2164-15-68 (PMC3922791; doi:10.1186/1471-2164-15-68)
Supplement: Additional file 2 — Fragmentase digestion protocol. PDF file of the protocol for fragmenting LR-PCR products with NEBNext® dsDNA Fragmentase™. [file 1471-2164-15-68-S2.pdf]

Clarke *et al. BMC Genomics* 2014, **15**:68 (Additional file 2)
http://www.biomedcentral.com/1471-2164/15/68

PROTOCOL

NEBNext® dsDNA Fragmentase™ digestion

If using this protocol, please cite:

Clarke AC, Prost S, Stanton J-AL, White WTJ, Kaplan ME, Matisoo-Smith EA, The Genographic Consortium: **From cheek swabs to consensus sequences: an A to Z protocol for high-throughput DNA sequencing of complete human mitochondrial genomes.** *BMC Genomics* 2014, **15**:68.

For updates to this protocol, please visit http://www.clarkeresearch.org/AtoZ.html

Background

NEBNext® dsDNA Fragmentase™ generates dsDNA breaks in a time-dependent manner, producing 100–800 bp fragments depending on incubation time [1].

The incubation time required to generate fragments of a given size range is affected by both the length of the starting molecules and GC-content, with longer molecules and those with a higher GC-content requiring a longer incubation time. We have also observed some variation in enzyme activity across tubes of Fragmentase supplied to us. For these reasons it is necessary to optimize digestion time for a given template and tube of Fragmentase.

In addition, we have observed that Fragmentase reactions are highly sensitive to variation in reaction composition and incubation time, and that it is important to control these variables to ensure consistent, reproducible results.

The following protocol is designed to supplement the NEB Application Note for Fragmentase [1]. All reaction components are as described in the Application Note.

Materials

Where a supplier is mentioned, this represents the supplier we have used; other suppliers' products may produce comparable results.

Reagents

All reagents should be an appropriate molecular biology grade.

- NEBNext® dsDNA Fragmentase™ (NEB, cat. no. M0348L)
- 0.5 M EDTA
- Agencourt® AMPure® XP (Beckman Coulter, cat. no. A63881)
- DNA 7500 Kit (Agilent, cat. no. 5067-1506)

Equipment

- 96-well cooler rack (e.g., Eppendorf, cat. no. 3881 000.015)
- Agencourt® SPRIPlate® Super Magnet Plate (Beckman Coulter, cat. no. A32782)
- Agilent 2100 Bioanalyzer

Reagent and equipment setup

Thermal cycler setup for Fragmentase incubation. A thermal cycler is ideal for controlling the temperature and duration of the incubation step. The 'PCR' program should include three steps. The first is step is 4°C for 1 min. This step ensures that reactions do not begin until all tubes are loaded, and that there are no effects from the ambient temperature of the block. The second step is 37°C for the desired incubation time [1], and the third step is a hold at 4°C. Do not use the hot lid when incubating Fragmentase reactions as this could lead to inconsistent results.

Procedure

Fragmentase digestion

1. Place the required number of 0.2 mL tubes or a 96-well plate on the cooler rack.

   ▲ CRITICAL STEP 8- or 12-well strip tubes can be used but it is often difficult to vortex these without the caps detaching. Ice can be used in place of a cooler rack, but heat transfer will be less efficient. It is also difficult to work with individual 0.2 mL tubes on ice.

2. Thaw and vortex the 10× reaction buffer and BSA.
3. Prepare a master mix of the 10× reaction buffer and BSA. The water can also be included in the master mix if the same volume of water is being used in all reactions.
4. Vortex the master mix.
5. Dispense aliquots of the master mix into the tubes/plate.
6. If not included in the master mix, add the required volume of water to the tubes/plate.
7. Add the required volume of template to the tubes/plate and incubate for 5 min.
8. Vortex the Fragmentase.
9. Add the required volume of Fragmentase to the tubes or plates.
10. Working quickly, close the caps or seal the plate.
11. Briefly remove the tubes/plate from the cooler rack and vortex thoroughly. Spin the reactions to the bottom of the wells using a centrifuge. Ensure the reactions are kept in the cooler rack whenever they are not being vortexed or in the centrifuge.
12. Start the incubation program on the thermal cycler, and once the block has reached 4°C insert the tubes/plate.
13. Once the 37°C incubation is complete and the thermal cycler black has cooled to 4°C, immediately remove the tubes/plate to the cooler block.

14. Working quickly, stop the reactions by the addition of 5 µL 0.5 M EDTA. Mix the reactions thoroughly by pipetting up and down. If a 96-well plate is being used, the EDTA can be dispensed from a 8- or 12-strip reservoir to speed up the process. As soon as the EDTA has been added the samples should be purified.

Fragmentase reaction purification

15. Purify the Fragmentase reactions using either standard AMPure XP, or if small fragments are to be removed, with the PEG–Bead Solution made in Additional file 3. Use 1.8 volumes of AMPureXP/PEG–Bead solution for each reaction (e.g., for a 20 µL Fragmentase reaction with 5 µL EDTA add 45 µL AMPureXP/PEG–Bead Solution). Purifications can be carried out exactly as described in Steps 5–8 of Meyer *et al.* [2]. Elute the fragments in 20 µL 10 mM Tris.

Bioanalyzer 2100 analysis of fragmented products

16. To visualise the digestion results, the Fragmentase reactions (or a subset thereof) can be run on the Bioanalyzer 2100 using a DNA 7500 chip according to the manufacturer's instructions.

    ▲ CRITICAL STEP Fragmentase reactions must be purified prior to being run on the Bioanalyzer 2100 because components of the Fragmentase reaction lower the surface tension in the chip wells and cause the samples to cross-contaminate when the chip is vortexed.

Acknowledgements

For technical assistance with this protocol we thank Aaron Jeffs (University of Otago, Dunedin, New Zealand).

References

1. New England Biolabs: **Fragmentation of RT-PCR and PCR Products with NEBNext dsDNA Fragmentase (M0348).** https://www.neb.com/protocols/1/01/01/fragmentation-of-rt-pcr-and-pcr-product-with-nebnext-dsdna-fragmentase-m0348.
2. Meyer M, Stenzel U, Hofreiter M: **Parallel tagged sequencing on the 454 platform.** *Nat Protoc* 2008, **3:**267–278.
