# Supplementary material for: From cheek swabs to consensus sequences: an A to Z protocol for high-throughput DNA sequencing of complete human mitochondrial genomes
Source: BMC Genomics. 2014 Jan 25;15:68. doi: 10.1186/1471-2164-15-68 (PMC3922791; doi:10.1186/1471-2164-15-68)
Supplement: Additional file 3 — Size selection protocol. PDF file of the protocol for DNA size selection using AMPure XP-derived PEG–Bead solution. [file 1471-2164-15-68-S3.pdf]

PROTOCOL

DNA size selection using AMPure XP-derived PEG–Bead solution

If using this protocol, please cite:

Clarke AC, Prost S, Stanton J-AL, White WTJ, Kaplan ME, Matisoo-Smith EA, The Genographic Consortium: **From cheek swabs to consensus sequences: an A to Z protocol for high-throughput DNA sequencing of complete human mitochondrial genomes.** *BMC Genomics* 2014, **15**:68.

For updates to this protocol, please visit http://www.clarkeresearch.org/AtoZ.html

Background

Standard AMPure XP is used to remove DNA fragments smaller than 100 bp. This cut-off can be increased or decreased (e.g., up to 300 bp, down to 50 bp) by isolating the beads from AMPure XP and resuspending them in PEG–Bead Solution containing polyethylene glycol (PEG) 8000. It is the concentration of PEG in the solution that defines the size cut-off, with increasing PEG concentration meaning a lower cut-off. Fragments up to 50 bp can be removed using a PEG concentration of approx. 30% (w/v) and up to 300 bp with a PEG concentration of approx. 10% (w/v). The relationship between fragment cut-off and PEG concentration is not linear however, with concentrations close to 10% having much larger effects than those close to 30%.

The optimum PEG concentration can be determined by trialling concentrations at 5% increments, and then narrowed down to 0.5–1.0% increments. The template that will ultimately be sequences can be used for testing, or alternatively a DNA ladder can be used as this makes it much easier to visualise the effects of varying PEG concentration. If using a ladder, do not use NEB ladders because the fragments are modified in a way that causes fragments of all sizes to be retained (Sebastian Lippold, pers. comm.) Both Fermantas and Invitrogen ladders are OK to use. Results can be visualised on an agarose gel (ladders only) or using the Bioanalyzer 2100.

Materials

Where a supplier is mentioned, this represents the supplier we have used; other suppliers' products may produce comparable results.

Reagents

All reagents should be an appropriate molecular biology grade.

- Agencourt® AMPure® XP (Beckman Coulter, cat. no. A63881)
- TET buffer (10 mM Tris–HCl, 1 mM EDTA, pH = 8.0, with 0.05% (v/v) Tween® 20)
- 50% (w/v) polyethylene glycol 8000 solution (Sigma-Aldrich, cat. no. 89510-250G-F)
- 5 M sodium chloride
- 10× TE buffer (100 mM Tris–HCl, 10 mM EDTA, pH = 8.0)
- Tween® 20 (Sigma-Aldrich, cat. no. P1379-100mL)

Equipment

- Agencourt® SPRIStand® – Magnetic 6-tube stand (Beckman Coulter, cat. no. A29182)

Reagent and equipment setup

PEG–Bead Solution. PEG–Bead Solution contains 10–30% (w/v) PEG 8000, 1M NaCl, 1 × TE (10 mM Tris, 1 mM EDTA) and 0.05% (v/v) Tween 20. The PEG–Bead Solution is added to the beads at Step 5 below. The PEG–Bead Solution is based on that described in Fisher *et al.* [1].

Procedure

Isolation of AMPure XP beads ● TIMING 20 min

1. Ensure AMPure XP beads are fully resuspended. Add the desired volume of AMPure XP (up to 1.5mL) to a 1.7 mL microtube.
2. Place tube(s) in the 6-tube SPRIStand and incubate for 10 min at room temperature. The buffer should become clear.
3. Carefully remove the buffer, taking care not to disturb the beads.
4. While still in the SPRIStand, wash the beads by adding 500 µL TET buffer. Incubate for 2 min and then completely remove the TET without disturbing the beads. Repeat this step.

Bead resuspension in PEG buffer

5. Remove the tube(s) from the SPRIStand and resuspend the beads in the desired PEG–Bead Solution. Use same volume of PEG–Bead Solution as the amount of AMPure XP originally used (e.g., if beads were isolated from 1.5 mL AMPure XP then resuspend the beads in 1.5 mL PEG–Bead Solution). Vortex the solution to ensure the beads are completely resuspended. The PEG–Bead Solution is stable at 4°C for at least two months or the expiry date of the AMPure XP, whichever is earlier.

Purification

6. Purify the DNA ladder or other DNA samples using the PEG–Bead Solution. Use 1.8 volumes of PEG–Bead solution for each reaction (e.g., for a 20 µL ladder solution add 36 µL PEG–Bead Solution). Purifications can be carried out exactly as described in Steps 5–8 of Meyer *et al.* [2]. Elute the fragments in 20 µL 10 mM Tris.

Bioanalzyer 2100 analysis of fragmented products

7. To visualise the results from a ladder trial, the eluate can be run on an agarose gel. Trials containing more complex mixtures of fragments (e.g., products from a Fragmentase reaction) should be run on the Bioanalyzer 2100 using a DNA 7500 chip according to the manufacturer's instructions.

Acknowledgements

For technical assistance with this protocol we thank Sebastian Lippold (Max Planck Institute for Evolutionary Anthropology, Leipzig, Germany).

References

1. Fisher S, Barry A, Abreu J, Minie B, Nolan J, Delorey TM, Young G, Fennell TJ, Allen A, Ambrogio L, *et al*: **A scalable, fully automated process for construction of sequence-ready human exome targeted capture libraries.** *Genome Biology* 2011, **12:**R1.
2. Meyer M, Stenzel U, Hofreiter M: **Parallel tagged sequencing on the 454 platform.** *Nat Protoc* 2008, **3:**267–278.
