# Supplementary material for: From cheek swabs to consensus sequences: an A to Z protocol for high-throughput DNA sequencing of complete human mitochondrial genomes
Source: BMC Genomics. 2014 Jan 25;15:68. doi: 10.1186/1471-2164-15-68 (PMC3922791; doi:10.1186/1471-2164-15-68)
Supplement: Additional file 4 — Bioinformatics pipeline. PDF file of the bioinformatics protocol. [file 1471-2164-15-68-S4.pdf]

Clarke *et al. BMC Genomics* 2014, **15:**68 (Additional file 4)
http://www.biomedcentral.com/1471-2164/15/68

PROTOCOL

Bioinformatics Pipeline

If using this protocol, please cite:

Clarke AC, Prost S, Stanton J-AL, White WTJ, Kaplan ME, Matisoo-Smith EA, The Genographic Consortium: **From cheek swabs to consensus sequences: an A to Z protocol for high-throughput DNA sequencing of complete human mitochondrial genomes.** *BMC Genomics* 2014, **15:**68.

For updates to this protocol, please visit http://www.clarkeresearch.org/AtoZ.html

Mapping Pipeline

The mapping pipeline is a Unix shell script that runs a series of other programs to transform a set of raw-read input files into a variety of useful output files.

The input to the pipeline is the immediate result of 454 sequencing: an .sff file containing all the short reads for all individuals. (Note that other sources of reads, such as Illumina GA sequencing, can be handled with small modifications to the script.) You also need a text file describing the tag sequences used. Each line in this file should contain a tag sequence, followed by one or more spaces or tabs, followed by the label to use for the corresponding individual. The final output of the pipeline consists of:

1. Per-individual consensus sequences as separate files in FASTA format in the subdirectory "consensus" (which will be created if it doesn't yet exist), and as separate sequences in the file all_consensus.fasta
2. A PostScript-format file, coverage.ps, containing coverage plots for every individual
3. A file snps_for_haplogrep.txt, containing SNPs for each individual in the format expected by HaploGrep (http://haplogrep.uibk.ac.at/). This web service can be used to infer the probable haplogroup of each individual based on the SNPs observed.

Some components of the pipeline, such as bwa and samtools, are C programs that need to be compiled before you can start using the pipeline for the first time. The first time that the pipeline is run, these programs will be compiled automatically; this will take a few minutes. If you already have versions of these programs installed, compilation will be skipped and the existing versions will be used instead.

Your computer must have the Perl and Python scripting languages installed in order to run the pipeline. If you do not have bwa or samtools installed already, you will also need a C++ compiler for the initial compilation step described above. All three programs (Perl, Python, and a C++ compiler) come preinstalled on modern versions of Linux and Mac OS X.

To run the pipeline, open a UNIX shell prompt or Terminal window and ensure that you have changed to the directory containing your .sff and tag files using the *cd* command. Then just type the path to the pipeline script, and provide the name of the .sff file and tag setup files, in that order, as parameters:

```
cd /path/to/your/data
/path/to/pipeline/mapping_pipeline.sh mydata.sff mytags.txt
```

By default, the pipeline uses the Revised Cambridge Reference Sequence of the human mitochondrial genome as the reference sequence for mapping. If you wish to use a different reference sequence, provide its filename as a third parameter when running the pipeline:

```
cd /path/to/your/data
/path/to/pipeline/mapping_pipeline.sh mydata.sff mytags.txt myrefseq.fasta
```

If you have added /path/to/pipeline to your $PATH, you can begin the second command with just "mapping_pipeline.sh" instead of the full "/path/to/pipeline/mapping_pipeline.sh". If you use the "bash" shell – the default command shell in most Linux and Mac OS X installations – you can add to your $PATH by using:

```
export PATH="$PATH":/path/to/pipeline
```

You only need to run this command once per terminal session; if you add it to the end of the .profile login script in your home directory, which gets run automatically whenever you log in, you won't need to type it again.

The pipeline script has been tested under Linux and Mac OS X, and should run without modification on any Unix system. Although it will not run on a Windows system, the individual commands are easy to interpret and corresponding commands can be entered manually once all the necessary C programs have been compiled.

If you need to run the pipeline script on multiple .sff files, ensure that each is in a different directory to prevent output files from earlier runs being overwritten. It is not necessary to have multiple copies of the pipeline directory itself.

The pipeline comes bundled with the following pre-existing standalone tools:

- Samtools: http://samtools.sourceforge.net/
- BWA: http://bio-bwa.sourceforge.net/
- sff_extract_0_2_13.py: http://bioinf.comav.upv.es/_downloads/sff_extract_0_2_13
- TagCleaner: http://tagcleaner.sourceforge.net/

It also includes the following new scripts, which can be used standalone:

- make_coverage_plots.pl
- get_ranges_from_coverage.pl
- put_snps_on_one_line.pl
- nuntag.pl (a replacement for the untag program from https://bioinf.eva.mpg.de/pts/, which can be difficult to get working)

All four scripts can be run separately, and can take options to control their behaviour; running them with -h or --help will explain the details.

Recommended Additional Software tool to assess read quality

- FastQC: http://www.bioinformatics.babraham.ac.uk/projects/fastqc/

Inside the Pipeline Script

The initial section of the pipeline script performs setup work: reading the names of the .sff file, tag setup file and, optionally, the reference sequence file from the command-line parameters supplied by the user; determining where the pipeline directory is; and compiling the samtools and bwa programs if they do not appear to exist already. The beginning of the bioinformatics pipeline proper is indicated by the comment line:

```
# BIOINFORMATICS BEGINS HERE
```

1. In the first step the 454 .sff file is converted to fastq, a version of a fasta file that also saves quality scores. Fastq is more commonly used in downstream analysis than .sff files.

```
sff_extract_0_2_13.py -c "$SFFFILE" --fastq -o sequencingfile
```

2. In the second step the reads are binned according to their barcodes. If you did not use a barcoding system, just leave this step out.

```
nuntag.pl -t "$TAGFILE" -o %N.raw.fastq -w mismatched_tags.fastq -u
    unrecognised_5p_tags.fastq sequencingfile.fastq
```

Alternative Steps 1 and 2

Our pipeline also allows you to use untag, the original script that is implemented in the PTS method. However, this script can be very difficult to install and therefore we implemented our own Perl script, nuntag.pl, and use it by default.

1. If you have untag already installed, you can use it instead:

```
untag -t"$TAGFILE" -w weird.fas -u untagged.fas --sff -o %N.untagged.sff "$SFFFILE"
```

2. If untag is used then the conversion from sff to fastq is performed after the sorting step.

```
#Convert sff file into fastq
for file in *.untagged.sff;do
    sff_extract_0_2_13.py $file --fastq -o `basename $file .untagged.sff`.raw
done
```

3. In the third step all primer sites are removed. Since we use two sets of primer pairs, we have to run this function twice, for each primer pair separately. We specify the five last bases at the 3' end of the forward primers and the first five bases at the 5' end of the reverse primers for the pattern search. The script is looking for these patterns within a range specified with -trim_within from the each end for of the reads. This trim_within function allows detecting partial priming sites.

```
# Remove primer sites
for file in *.raw.fastq;do
    tagcleaner.pl -fastq $file -tag5 TTAAC -mm5 1 -tag3 CTAGT -mm3 1 -
      trim_within 26 -out `basename $file .raw.fastq`.pp1
done

for file in *.pp1.fastq;do
    tagcleaner.pl -fastq $file -tag5 TTACT -mm5 1 -tag3 GTTTT -mm3 1 -
      trim_within 25 -out `basename $file .pp1.fastq`.pr
done
```

4. This step simply indexes the reference sequence, so that it can be used for the mapping step (Step 6.2).

```
# Indexing the reference
bwa index -a is "$REFSEQ"
```

5. Creates a folder to store consensus sequences called in the Step 6 loop.

```
# Create directory for consensus sequences if it doesn't already exist
mkdir -p consensus
```

6.1. Starts a loop until Step 6.12. The loop includes mapping, raw data processing and creating consensus sequences for all files processed in Step 3.

```
for file in *.pr.fastq;do
```

6.2. Strip off the “.pr.fastq” suffix and store the rest in the variable ${bn}.

```
bn=`basename $file .pr.fastq`
```

6.3. In the first step within the loop, the reads are mapped against a reference sequence (by default, the included rCRS.fasta) using BWA with the bwasw function for long read data.

```
# Mapping
bwa bwasw "$REFSEQ" $file > ${bn}.sam
```

6.4. The .sam file is compressed using BGFZ compression and saved in a .bam file.

```
# Changes the mapping file (.sam) to a BGFZ compressed BAM file (.bam)
samtools view -bS ${bn}.sam > ${bn}.bam
```

6.5. Sorts the reads according to the reference sequence.

```
# Sort to coordinates from RefSeq
samtools sort ${bn}.bam ${bn}.sorted
```

6.6. Indexes nucleotide positions.

```
# Index sorted alignment
samtools index `basename $file .pr.fastq`.sorted.bam
```

6.7 (optional). As discussed above in the introduction, a realignment step might be appropriate. GATK (http://www.broadinstitute.org/gsa/wiki/index.php/Home_Page) is commonly used for this step. However, it cannot handle 454 data and was thus not included into the pipeline.

```
# Realignment
```

6.8. In this step, the *mpileup* function of samtools is used to create a summary file, which can then be used to make coverage plots (Step 7) or to get the ranges for the Haplogrep SNP input file (Step 9).

```
# Create coverage files
samtools mpileup -D ${bn}.sorted.bam > ${bn}.cov
```

6.9. Here we use the *mpileup* function and bcftools to call a consensus sequence and store it into a fastq file. Note that this version will call ambiguity codes in the presence of heteroplasmy. If you prefer not to produce ambiguity codes, please use the alternative below.

```
# Create consensus in fastq
samtools mpileup -uf "$HERE/rCRS.fasta" ${bn}.sorted.bam | bcftools view -cg - |
    vcfutils.pl vcf2fq > ${bn}.fq
```

6.9 (alternative). Here we use samtools 0.1.16 to call a consensus sequence that does not include ambiguity codes, by forcing it to call only the major nucleotide in cases of conflicting reads. (Unfortunately the –N 1 function has been removed from Samtools 0.1.17 and up.)

```
# Create consensus in fastq without ambiguity codes
samtools16 pileup -N 1 -cf "$REFSEQ" ${bn}.sorted.bam | samtools.pl pileup2fq -D30
    > ${bn}.fq
```

6.10. Here we extract the first (consensus) sequence from the fastq file, rename it within the file to have the same name as the file basename, and save the result into a simple fasta file in the folder “consensus”.

```
# Get first sequence in fasta format and rename it
perl -pe 'exit if /^\+/; s/^\@.*/>'${bn}'/ if $. == 1' ${bn}.fq >
    consensus/${bn}_assembled.fasta
```

6.11. Last step of the loop.

```
done
```

7. In Step 7 coverage plots for all .cov files are saved into a PostScript file called coverage.ps.

```
# Make coverage plot
make_coverage_plots.pl *.cov > coverage.ps
```

8.1. In Step 8 we use samtools and bcftools to call SNPs. Step 8.1 starts the loop.

```
for file in *.sorted.bam;do
```

8.2. Strip off the “.sorted.bam” suffix and store the rest in the variable ${bn}.

```
bn=`basename $file .pr.fastq`
```

8.3. Samtools and bcftools are used to call SNPs in the mapping (compared to the reference sequence, here rCRS.fasta). Note that the –I command suppresses indel calling. In general, we would not recommend calling indels using 454 data.

```
# Call SNPs
samtools mpileup -Iuf "$REFSEQ" $file | bcftools view -bvcg -> ${bn}.raw.bcf
```

8.4. We wish to also apply a filter to retain only quality SNPs. The first step is to calculate the maximum allowed read depth, and store it in a file whose name ends with .flt.maxcov. For this we use twice the average coverage, as recommended in the samtools documentation.

```
# Filter SNPs
estimate_read_depth_for_filtering.pl < ${bn}.cov > ${bn}.flt.maxcov
```

8.5. We now apply the filter, using this maximum read depth.

```
bcftools view ${bn}.raw.bcf | vcfutils.pl varFilter -D`cat ${bn}.flt.maxcov` >
    ${bn}.flt.bcf
```

8.6. done stops the SNP calling loop.

```
done
```

9.1. In Step 9 we use a Perl script to save the called SNPs into a HaploGrep SNP input file.

```
# Make empty HaploGrep SNP input file
put_snps_on_one_line.pl > snps_for_haplogrep.txt
```

9.2. Create the rest of the input file for HaploGrep:

```
#Create Haplogrep input SNP file
for file in *.flt.bcf;do
        bn=`basename $file .flt.bcf`
        put_snps_on_one_line.pl --no-header -r `get_ranges_from_coverage.pl -t 1
            ${bn}_cov.txt` $file >> snps_for_haplogrep.txt
done
```

This HaploGrep SNP input file can then be used to call haplotypes using the web tool HaploGrep (http://haplogrep.uibk.ac.at/).

10. Join all consensus sequences into a single fasta file:

```
# Produce a single FASTA file containing all consensus sequences
cat consensus/*_cons.fasta > all_consensus.fasta
```

Data quality check

The mapped data quality can be assessed using the software tool FastQC (http://www.bioinformatics.babraham.ac.uk/projects/fastqc/), which has not been provided with the pipeline bundle.

Coverage Plots

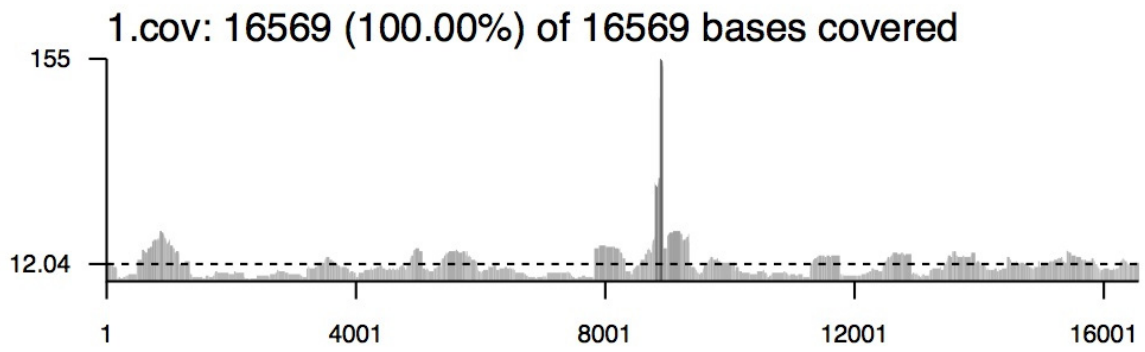

Figure S1 Coverage Plot. The plot shows the coverage of each position of the reference in the mapping. The density is shown in different colors or shades of grey, by default ranging from light (low coverage) to dark (high coverage). The mean coverage is shown by a dotted line and its value on the left side. The header displays the name of the mapping and the count and % of the positions covered by at least one read.
